# Association between patient safety culture and professional quality of life among nursing professionals

**DOI:** 10.1590/1980-220X-REEUSP-2023-0359en

**Published:** 2024-07-08

**Authors:** Edenise Maria Santos da Silva Batalha, Elisabete Maria das Neves Borges, Marta Maria Melleiro

**Affiliations:** 1 Universidade do Estado da Bahia, Departamento de Ciências da Vida, Salvador, BA, Brazil.; 2 Escola Superior de Enfermagem do Porto, Porto, Portugal.; 3 Universidade de São Paulo, Escola de Enfermagem, São Paulo, SP, Brazil.

**Keywords:** Patient Safety, Personal Satisfaction, Burnout, Psychological, Stress Disorders, Traumatic, Nursing, Seguridad del Paciente, Satisfacción Personal, Agotamiento Psicológico, Trastornos de Estrés Traumático, Enfermería

## Abstract

**Objective:**

To analyze the association between patient safety culture and professional quality of life in nursing professionals.

**Method:**

Correlational study carried out in a hospital in Salvador, Bahia, Brazil, with 180 participants. The data were collected through the Hospital Survey on Patient Safety Culture and Professional Quality of Life Scale and analyzed with correlation tests.

**Results:**

The use of the Quality of Professional Life model, which encompasses Compassion Satisfaction, Burnout and Traumatic Stress, showed that a better assessment of the safety culture was negatively associated with Burnout. Regarding the dimensions of culture, better evaluations of the general perception of safety, teamwork and staffing were negatively associated with Burnout and Traumatic Stress. Higher Burnout was negatively associated with better handoffs and greater Traumatic Stress was positively associated with error communication.

**Conclusion:**

Higher levels of Burnout were associated with worse perception of safety culture and worse teamwork evaluations; staffing and general perception of safety were associated to a higher level of Burnout and Traumatic Stress, which emphasizes the importance of investment in these areas.

## RESUMEN

**Objetivo:**

Analizar la asociación entre la cultura de seguridad del paciente y la calidad de vida profesional de los profesionales de enfermería.

**Método:**

Estudio correlacional realizado en un hospital de Salvador, Bahía, Brasil, con 180 participantes. Los datos fueron recolectados a través de los instrumentos *Hospital Survey on Patient Safety Culture* y *Professional Quality of Life Scale* y analizados con pruebas de correlación.

**Resultados:**

El uso del modelo de Calidad de Vida Profesional, que engloba la Satisfacción por compasión, el *Burnout* y el Estrés Traumático, demostró que una mejor evaluación de la cultura de seguridad se asoció negativamente con el *Burnout*. Respecto a las dimensiones de la cultura, mejores evaluaciones de la percepción general de la seguridad, el trabajo en equipo y la dotación de personal se asociaron negativamente con el *Burnout* y el estrés traumático. Un mayor *Burnout* se asoció negativamente con un mejor cambio de turno y un mayor estrés traumático se asoció positivamente con la comunicación de errores.

**Conclusión:**

Mayores niveles de *Burnout* se asociaron con peor percepción de la cultura de seguridad y peores evaluaciones del trabajo en equipo; la dotación de personal y la percepción general de seguridad se asociaron con un mayor nivel de *Burnout* y Estrés Traumático, lo que enfatiza la importancia de la inversión en estas áreas.

## INTRODUCTION

Strengthening a positive patient safety culture, based on a systemic view of health services, prioritizing incident prevention and learning, is essential to guarantee quality of care. This culture is recognized as one of the main recommendations for mitigating adverse health events. Consequently, its evaluation has been widely used in health institutions, enabling the identification of vulnerable cultural dimensions and a diagnosis of intervention needs, as well as the direction of actions for improvement^(1,2)^.

Investment in patient safety culture through evaluation and intervention proposals requires the understanding that health activities are essentially performed by individuals and that their physical and psychological health is a crucial factor for the development and success of safety policies. Furthermore, it is essential to understand that the work environment and its safety level may influence worker health and well-being. Therefore, strengthening patient safety culture requires integrating this area with other organizational aspects and worker health in particular. The analysis of this relationship may thus clarify how other areas can be enhanced, jointly leading to positive results for patients and professionals.

In this context, a reflection on the work carried out by nursing professionals is necessary, as they are the largest health workforce and provide direct and continuous care to patients, coordinating the work of other professionals and ensuring assistance^(3)^. However, due to the inherent characteristics of their work process and working conditions, these workers are exposed to physical and psychological loads that may damage their health and well-being, possibly impacting assistance^(4)^.

In their daily work, these professionals are exposed to sources of distress, either due to dealing with the paradoxes of life and death, health, and illness or due to work environment conditions. These sources of distress include the lack of physical structure and material resources, associated with work overload and managerial neglect of these issues, in addition to patient mortality and uncommitted team members^(5)^. Furthermore, these workers are often subjected to high-intensity work, precarious working conditions, and unsafe clinical processes and procedures^(3)^.

Despite the negative aspects, work provides pleasurable experiences, as well as professional and personal satisfaction. In this sense, nursing professionals find pleasure in their work through recognition from patients, the possibility of recovery, the improvement of clinical conditions, and maintaining positive relations with the team^(5)^.

Based on the duality of positive and negative feelings generated and amplified by work centered on providing care, psychologist Beth Hudnall Stamm proposed a model named “Professional Quality of Life”, incorporating both the positive aspect, Compassion Satisfaction, and the negative one, Compassion Fatigue, divided into Burnout and Secondary Traumatic Stress^(6)^.

Compassion satisfaction refers to professional pleasure when adequately performing tasks and relates to being able to help others and their ability to contribute to the work environment. In turn, the first aspect of compassion fatigue concerns feelings such as exhaustion and frustration, typical of Burnout, associated with feelings of hopelessness and difficulties in coping with work or working effectively. The second aspect, secondary traumatic stress, is related to exposure to extreme stressful and traumatic events at work, being characterized by fear and care-related trauma. Negative effects may include sleep difficulties due to fear, intrusive images, or avoidance of traumatic memories^(6)^.

Given the consequences of these aspects for workers, health organizations and patients, research has been using professional quality of life as a model^(7–9)^. However, there are still few studies that address the relationship between professional quality of life and patient safety culture in Brazilian hospitals. Analyzing this relationship is essential for understanding the association between these factors and for designing combined strategies arising from health quality, patient safety, and worker health policies.

Given these considerations, this investigation aimed to analyze the association between patient safety culture and professional quality of life of nursing workers. Given the characteristics of the phenomena, it tested also the hypothesis that better safety culture assessments would be positively associated with compassion satisfaction and negatively associated with Burnout and secondary traumatic stress.

## METHOD

### Design of Study

This is an exploratory, cross-sectional, correlational study with a quantitative approach.

### Local

The research was carried out in a general public hospital in the metropolitan region of Salvador, in the state of Bahia, Brazil. The institution, part of the Bahia State Health Department’s Network, comprises 640 available beds and is considered a large tertiary care institution with a high-complexity profile. It is included in the care network of the Eastern Health Macroregion of the State of Bahia, providing outpatient and inpatient care, in addition to emergency and urgent hospital care. This hospital is a reference center in neurology, in addition being a reference for digestive hemorrhage, nephrology, pediatrics, clinical medicine, oral and maxillofacial surgery, general surgery, neurosurgery, pediatric and neonatal surgery, high-risk maternity, among other specialties.

Given the variety of services offered by the hospital, the data collection was chosen to be structured in the Medical-Surgical and Maternal-Child hospitalization areas. Thus, collection took place in the following units: Medical Clinic with 96 beds; Surgical Clinic with 107 beds; Adult Intensive Care Unit with 20 beds; Neonatal Intensive Care Unit with 17 beds and Obstetrics with 60 beds. These units totaled, during the collection period, 300 active available beds.

### Population and Selection Criteria

The study population comprised nurses, technicians, and nursing assistants who worked in the units where data collection was performed, totaling 528 nursing workers.

Convenience sampling was employed and the exclusion criteria were workers with less than 6 months at the institution, who were on vacation, leave and/or absence from service at the time of data collection, and who carried out exclusively administrative activities. After applying the exclusion criteria, the number of eligible workers was 420.

### Sample Definition

As this is a correlational study, the sample size assessment considered the correlation between the variables and adopted a correlation coefficient ≥ 0.25 as the minimum effect size, a signiﬁcance level of 5%, and a test power of 90%^(10)^. Based on these parameters, the sample included 180 nursing workers.

### Data Collection

Data collection took place from January to March 2020 through three questionnaires. The first, called socioeconomic and professional questionnaire, was prepared by the authors and aimed at characterizing the sample through 12 questions: eight multiple choice questions (e.g., gender and professional category) and four open for free completion (e.g., age and professional experience). The second was Hospital Survey on Patient Safety Culture (HSOPSC), by the Agency for Healthcare Research and Quality (AHRQ)^(11)^, translated and validated for Brazil^(12)^, and the third was the Professional Quality of Life Scale 4 *–* ProQol IV^(13)^, translated and validated for Brazil^(14)^.

The HSOPSC presents 42 assertions distributed into 12 dimensions, namely: Dimension (D)1: Teamwork Within Units; D2: Overall Perceptions of Safety; D3: Nonpunitive Response to Error; D4: Staffing; D5: Organizational Learning—Continuous Improvement; D6: Supervisor/Manager Expectations & Actions Promoting Safety; D7: Communication Openness; D8: Feedback and Communication About Error; D9: Frequency of Event Reporting; D10: Hospital Handoffs and Transitions; D11: Hospital Management Support for Patient Safety; D12: Teamwork Across Hospital Units. Furthermore, it evaluates the patient safety score and the number of events reported by workers in the last 12 months. This questionnaire includes a socio-psychological scale with five levels of psychometric measures (ranging from Totally Disagree to Totally Agree and from Never to Always), in which, for analysis purposes, measure 1 is considered the worst evaluation and 5, the best evaluation.

The Brazilian version of ProQol IV consists of 28 questions distributed across the three components. The items in the Compassion Satisfaction subscale address the benefits of work, and its questions deal with pride, the feeling of being able to make a difference in people’s lives, enthusiasm, and satisfaction in caring for others. While the secondary traumatic stress subscale refers to negative factors of professional activity, addressing tension, stress, and trauma related to working with people in distress. Therefore, it deals with the harmful effects of secondary exposure to stressful events. Finally, the Burnout subscale covers aspects related to emotional exhaustion, the feeling of lack of energy and weariness. The ProQol IV also adopts a scalar measurement in five intensity levels ranging from rarely to almost always, with scores from 1 to 5.

The workers were invited to take part in the research in their workplace, in person, and the period for returning the questionnaire was established. This period varied depending on the availability of participants. The workers were given the choice of responding when invited to the survey or of taking the questionnaire with them and returning it to the researchers at another time. The mean time to answer the three questionnaires was 20 to 25 minutes. The collection was carried out by the main researcher with the assistance of three nursing students from a state university in Bahia. All clarifications about the research were provided and doubts were resolved by the researchers as soon as presented by the participants.

### Data Analysis and Treatment

The data were organized and stored in Microsoft Excel spreadsheets and the validation of the questionnaires to carry out the analyses was based on criteria established by the authors of the original instruments.

The sociodemographic and professional data were analyzed using descriptive statistics through the distribution of absolute and relative frequencies and numerical variables analyzed by frequencies in class intervals and by calculating the mean and its standard deviation.

To achieve the objective of this study and test its hypotheses, we used the *Spearman* Correlation Test. In this way, this test measured the association of each of the dimensions of patient safety culture, as well as total patient safety (calculated from the analysis of all dimensions), the patient safety score, and the number of events with compassion satisfaction, secondary traumatic stress, and Burnout.

Residual normality was assessed through the Shapiro-Wilk Test and homoscedasticity through Levene’s Test. A significance level of 5% was adopted (95% confidence level) for all tests. The statistical program R was used to carry out the analyses.

### Ethical Aspects

This study complied with the ethical principles involving research with human beings determined by Resolution No. 466/2012 of the National Health Council and was approved by the Research Ethics Committee (REC) of the School of Nursing of Universidade de São Paulo (Opinion no. 3.285.766, year 2019) and by the REC of the hospital – study setting (Opinion no. 3.731.330, year 2019). The participants were apprised through the Informed Consent Form, which was delivered in two identical copies signed by the responsible researcher and the participant. A copy was left with both parties, guaranteeing participation in an autonomous, conscious, free, and informed manner.

## Results

### Study Participants

The sample consisted of 180 nursing workers, 158 (87.8%) of whom were female, with a mean age of 40 years. The largest number was that of nursing technicians (n = 95; 52.8%) and the prevalent level of education was high school (n = 64; 35.8%). The majority (n = 62; 34.4%) worked in Surgical Clinics, had 10 to 14 years of professional experience (n = 47; 26.1%) and 6 months to 4 years of experience in the institution (n = 89; 50.0%).

The complete description of sociodemographic and professional data is found in Table 1.

**Table 1:** Characterization of study participants according to sociodemographic and professional variables – Salvador, BA, Brazil.

Sociodemographic and professional variables		N	%
**Sex**		180	100.0
	Female	158	87.8
	Male	22	12.2
**Age** (M = 40.0 SD = 8.4)		175	100.0
	19–28 years old	13	7.4
	29–38 years old	65	37.1
	39–48 years old	67	38.3
	Over 49	30	17.2
**Professional category**		179	100.0
	Nursing Assistant	11	6.1
	Nursing Technician	95	52.8
	Nurse	73	40.6
**Education**		179	100.0
	High School	64	35.8
	Undergraduate	49	27.4
	Postgraduate	47	26.3
	Master’s	19	10.6
**Workplace**		180	100.0
	Medical Clinic	37	20.6
	Surgical Clinic	62	34.4
	Adult ICU	36	20.0
	Neonatal ICU	27	15.0
	Obstetrics	18	10.0
**Professional experience** (M = 11.6 SD = 8.3)		180	100.0
	6 months – 4 years	41	22.8
	5–9 years	40	22.2
	10–14 years	47	26.1
	15–24 years	34	18.8
	Over 25	18	10.0
**Length of service** (M = 7.0 years SD = 6.6)		178	100.0
	6 months – 4 years	89	50.0
	5–9 years	31	17.4
	10–14 years	39	21.9
	Over 15 years	19	10.7

M: mean; SD: standard deviation; ICU: Intensive Care Unit.

### Association Between Patient Safety Culture and Professional Quality of Life

Compassion Satisfaction was not associated with patient safety culture.

Burnout showed a significant negative correlation with total patient safety with a correlation coefficient (r) of –0.287 and p-value of <0.001 and with safety grade (r = –0.191; p = 0.01), demonstrating that, in general, higher rates of Burnout were negatively associated with a better assessment of patient safety culture. Specifically, higher Burnout scores were negatively associated with five dimensions of patient safety culture: D1: Teamwork within units (r = –0.353; p < 0.001 ); D12: Teamwork across hospital units (r = –0.322; p ≤ 0.001); D2: Overall perceptions of safety (r = –0.306; p ≤ 0.001); D4: Staffing (r = –0.250; p ≤ 0.001); and D10: Hospital handoffs and transitions (r = –0.148; p ≤ 0.05).

Overall, patient safety culture was not associated with secondary traumatic stress. However, when it comes to specific dimensions, higher rates of secondary traumatic stress were also associated with worse evaluations of the following dimensions: D1: Teamwork within units (r = –0.207; p ≤ 0.01); D2: Overall perceptions of safety (r = –0.188; p ≤ 0.05); D4: Staffing (r = –0.193; p ≤ 0.01); and D12: Teamwork across hospital units (r = –0.147; p ≤ 0.01). Secondary traumatic stress was positively associated with a better score in dimension D8: Feedback and communication about error (r = 0.16; p ≤ 0.05).

**Figure 1:**
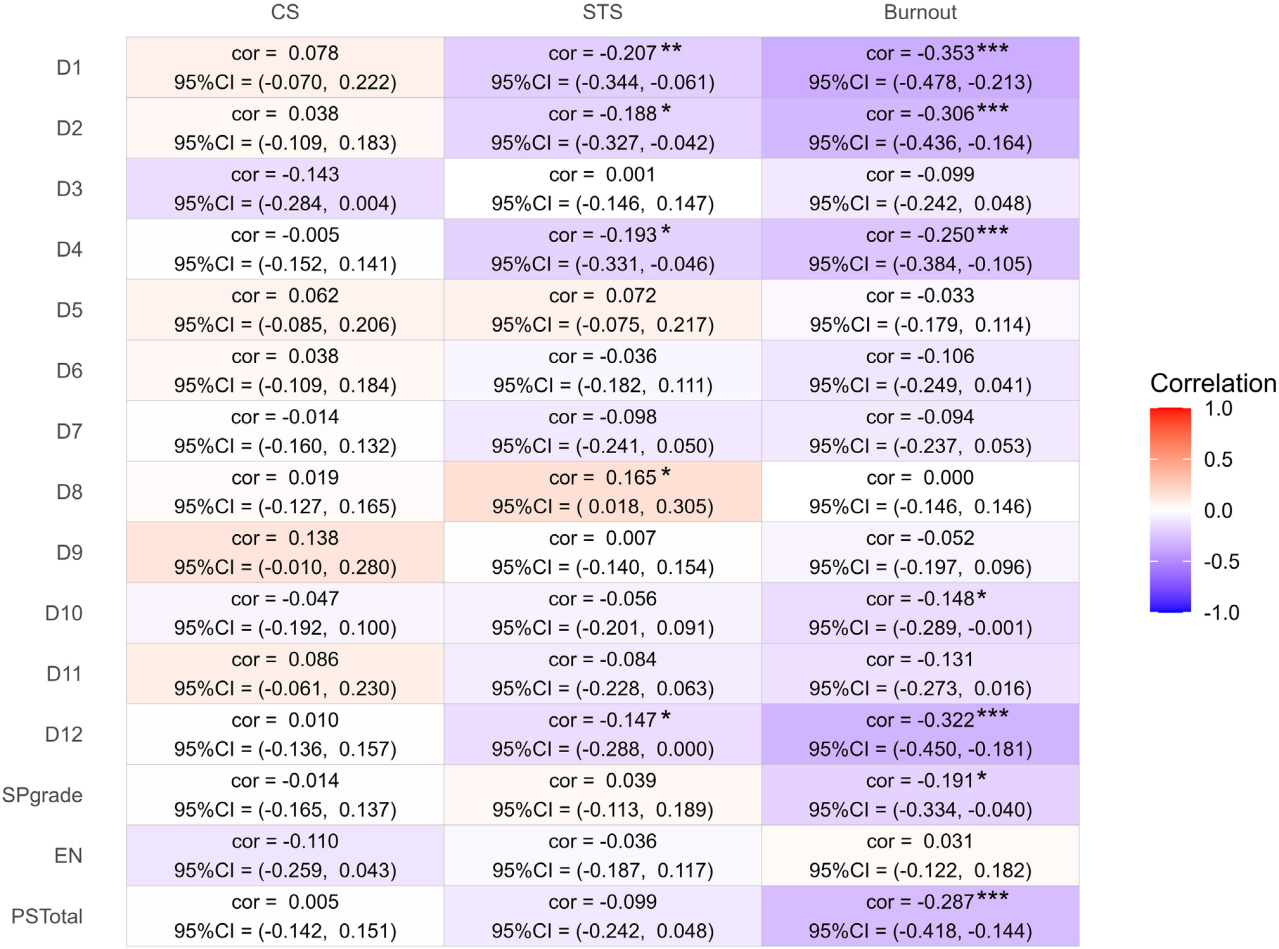
Analysis of correlations between the dimensions of Patient Safety Culture, Patient Safety Score, Number of Reported Events, and Total Patient Safety with Compassion Satisfaction, Stress Traumatic Secondary and Burnout – Salvador, BA, Brazil, 2020.

The complete results are shown in Figure 1. Figure 1 reveals, in addition to the values of the correlation coefficient, confidence interval and significance, the colors referring to the heat map: positive correlations are presented in shades in red and the negative ones are in shades of blue; the darker the color, the stronger the correlation between the variables. There is a predominance of the darker shade of blue in the relationship between Burnout and patient safety culture.

Cor: correlation; 95% CI: Confidence interval; *p-value <0.05; **p-value <0.01; ***p-value <0.001. D1: Teamwork Within Units; D2: Overall Perceptions of Safety; D3: Nonpunitive Response to Error; D4: Staffing; D5: Organizational Learning—Continuous Improvement; D6: Supervisor/Manager Expectations & Actions Promoting Safety; D7: Communication Openness; D8: Feedback and Communication About Error; D9: Frequency of Event Reporting; D10: Hospital Handoffs and Transitions; D11: Hospital Management Support for Patient Safety; D12: Teamwork Across Hospital Units. SPgrade: Patient safety grade; EN: Number of safety events reported in the last 12 months; PSTotal: Patient Safety Total; CS: Compassion Satisfaction and STS: Secondary Traumatic Stress.

## DISCUSSION

Patient safety culture was not associated with compassion satisfaction, a result that differed from a previous study carried out with nurses in Portugal, in which a significant positive association was found between compassion satisfaction and four dimensions of patient safety culture: Teamwork across hospital units; Overall perceptions of safety; Staffing; and Organizational learning—continuous improvement. Furthermore, in this study on the Portuguese context, patient safety score was positively associated with compassion satisfaction^(15)^. Aspects that may justify these findings include social and cultural issues, as Brazil and Portugal are different countries, with different configurations in relation to the health system, the organization of the nursing area, and the work process itself, which can influence in how satisfaction is conceived and developed in workers and how it relates to other organizational factors.

Although the hypothesis that compassion satisfaction would be positively associated with patient safety culture in this study was not confirmed, it is necessary to reflect on its importance. A study with 10,305 Korean nurses identified that compassion satisfaction has a mediating effect on the relationship between stress and Burnout and confirmed that, even in a stressful situation, a nurse experiencing compassion satisfaction can counterbalance the relationship between stress and Burnout, resulting in reduced Burnout^(16)^. Furthermore, studies have shown that compassion satisfaction has a significant negative association with burnout and secondary traumatic stress^(7,8,14)^, demonstrating the magnitude of its positive effect.

The negative association of Burnout with patient safety culture confirmed this study’s hypothesis and is congruent with the results found in the study in the Portuguese context^(15)^. Overall, patient safety culture was not associated with secondary traumatic stress. However, it was negatively associated with four dimensions of safety culture and positively associated with one of them, which partially confirms this study’s hypothesis. The Portuguese study found a negative association between traumatic stress and three dimensions: Staffing, Overall perceptions of safety, and Nonpunitive response to errors^(15)^.

The associations observed between Burnout and secondary traumatic stress and patient safety culture can be assessed by suggesting that we may be in the face of a cyclical relationship. Considering the characteristics of Burnout and secondary traumatic stress, nursing workers affected by these changes could be more prone to a lower compliance with quality standards and, therefore, to providing less safe care. In addition, they might present more difficulties in their relationships at work, evaluating patient safety culture as worse. Secondarily, since the work environment is unsafe for patients, these workers could suffer from the effects of a deficient safety culture and present higher levels of Burnout and secondary traumatic stress^(15,17)^.

Specifically in relation to Burnout and patient safety, a review study showed that higher levels of Burnout were associated with lower levels of patient safety culture, a lower frequency of notifications of safety incidents, higher rates of falls, healthcare-related infections, medication errors, and lapses in adherence to infection control. High levels of Burnout influenced the perception of pressure at work, which was negatively related to patient safety. Furthermore, a better work environment was directly associated to lower levels of Burnout, which was subsequently related to a higher level of patient safety and the mitigation of adverse events^(17)^.

Studies continue to point to the negative relationship between Burnout and safety culture, demonstrating that workers in situations of high demand at work and who experience Burnout are more likely to negatively evaluate patient safety culture^(18,19)^.

The specific dimensions that were negatively associated with Burnout and secondary traumatic stress were more influent on the evaluation of the components of compassion fatigue. In addition, workers with higher scores and, possibly, more changes in their well-being due to traumatic stress and/or Burnout scored more negatively in these aspects. Therefore, we emphasize the need for improving these dimensions, combined with prevention and mitigation of Burnout and secondary traumatic stress.

The Brazilian context faces a major challenge when it comes to improving patient safety culture in general. A scoping review analyzed 36 studies that used HSOPSC in hospitals in Brazil and showed that in 27 no dimensions were strengthened in the studied institutions^(1)^. This data shows that a positive patient safety culture in hospitals is far from being achieved and leads us to reflect that this weakened culture can cause more suffering in the work process.

Continuing education is highlighted as a strategy that can improve overall patient safety culture through specific training programs, the promotion of open communication in the work environment, the encouragement of reporting incidents, and the implementation of a non-punitive culture^(2)^.

The evaluation of teamwork within the unit and across hospital units stood out in its association with Burnout and secondary traumatic stress, demonstrating that, the more workers perceived teamwork as deficient, the higher they scored in Burnout and secondary traumatic stress.

Teamwork enables the production of better results in healthcare and increased job satisfaction and should be encouraged as one of the strategies for the increasing complexity of patient demands and of health organizations. The attributes of teamwork are communication, common goals, recognition of the work of all team members, interdependent actions, interprofessional collaboration, and patient-centered care^(20)^. In view of these findings, the need to invest in teamwork is emphasized, aiming to enhance its attributes in the work context.

Better staffing scores were also negatively associated with Burnout and traumatic stress. This result may be related to a known association between high workload environments and Burnout^(6,21)^. Proper sizing provides a more equitable work environment and provides more opportunities for the team to develop mutual support and harmonious relationships, with respect and social support among members. In line with this thought, a study showed that low social support may predispose to an increased risk of secondary traumatic stress^(22)^.

In the analysis of correlation strength, Burnout presented stronger negative correlation with patient safety culture, demonstrating that, with an increased level of Burnout, workers tended to reduce their positive evaluation of safety culture. These findings possibly relate to the characteristics of Burnout, since it is more progressive and continuous, resulting from stressful situations and conflict that can lead to lower work commitment, reduced empathic concern for patients, and bad feelings towards coworkers, oneself, and the profession^(6,21)^.

Burnout is associated with frustration and fatigue when work no longer meets expectations, generating a feeling of emptiness in which there is a significant rupture in professional identity, leading to decreased commitment and dissatisfaction, which affects their performance of work activities^(21)^. In contrast, traumatic stress arises from the possibility of experiencing empathy for suffering others, with the empathic process being a resource for healthy and compassionate involvement with people^(22)^. Furthermore, traumatic stress can be acute and abrupt, with, in most cases, a faster recovery than Burnout^(6)^.

The dimension feedback and error communication, which refers to workers receiving information on errors, feedback on changes, and discussing ways to prevent errors, showed a significant positive association with secondary traumatic stress. A study has shown that professionals who experience errors in their work process present trauma-related symptoms^(23)^. Although professionals recognize these symptoms, they state that errors are best dealt with through communication and discussion (debriefing*)* with the team. Furthermore, professionals involved in errors say that talking to colleagues, patients, and relatives about the error is also essential^(23)^. This analysis demonstrates the importance of communicating about errors as a fundamental principle for strengthening patient safety culture and professional well-being.

Although they have different characteristics and relate differently to patient safety culture, combined measures aimed at reducing Burnout and secondary traumatic stress are essential for promoting worker health and patient safety. The importance of these interventions is shown by studies demonstrating a significant positive association between these two aspects of compassion fatigue^(7,8,14)^. Therefore, implementable measures include conducting interventions based on meditation, breathing, and self-compassion exercises during work shifts^(9)^, which were shown to be effective in improving rates of compassion satisfaction and to reduce compassion fatigue.

Furthermore, specific strategies for the prevention and relief of Burnout become essential and must be employed at an individual, collective/group, and organizational level. Concerning individual strategies, workers can be encouraged to develop self-evaluation and self-knowledge, in addition to adopting healthier habits. In a collective/group level, strategies must be promoted to encourage mutual help, the communication of feelings and perceptions with the team, the construction of strong work collectives with mutual support. Interventions at the organizational level are centered on the opportunity for active participation of workers in work decisions and in the restructuring of tasks and working conditions, in order to make them attractive and rewarding for workers^(24)^.

Despite presenting significant contributions to the area of patient safety and worker health, this study has some limitations. The first refers to the fact that it was developed in a specific hospital setting, with a restricted sample of nursing workers, so the generalization of the results must be done with caution. The other concerns the fact that this study was developed only with the nursing team and thus does not present results referring to other professional categories.

Despite these limitations, this study is innovative, contributing to improvements in the care and management of nursing and health services, as it points to the need to restructure organizational safety policies for patient and worker health, with special emphasis on teamwork and adequate staffing, and the prevention and relief of Burnout.

## CONCLUSION

From the analysis of the association between patient safety culture and professional quality of life, it was found that compassion satisfaction was not associated with patient safety culture. Despite this, given its positive effects, compassion satisfaction should be encouraged among health professionals. When it comes to compassion fatigue, the negative relationship between Burnout and a better assessment of patient safety culture was confirmed, demonstrating that workers with higher levels of Burnout tended to evaluate as worse patient safety in the work unit and in the hospital as a whole, which highlights the need for interventions aimed at strengthening safer care and Burnout prevention and relief, combining strategies at the individual, collective and organizational levels. Worse evaluations in the dimensions relating to teamwork, staffing, and overall perception of safety were associated with a higher level of Burnout and traumatic stress, emphasizing the importance of higher investment in these areas.

Given these considerations, this study made it possible to verify how these aspects are related and how feasible it is to evaluate them in a combined way to implement improvements that benefit both patients and professionals and, consequently, health services.

Finally, this investigation is believed to possibly open paths for new research, including longitudinal studies aimed at monitoring and analyzing aspects and their association over time, allowing for a deeper understanding of this relationship and the construction of new intervention proposals.
