# Supplementary material for: Association between patient safety culture and professional quality of life among nursing professionals
Source: Rev Esc Enferm USP. 2024 Jul 8;58:e20230359. doi: 10.1590/1980-220X-REEUSP-2023-0359en (PMC11268136; doi:10.1590/1980-220X-REEUSP-2023-0359en)
Supplement: Translation

# Translation: Associação entre cultura de segurança do paciente e qualidade de vida profissional de trabalhadores de enfermagem

**DOI:** 10.1590/1980-220X-REEUSP-2023-0359pt

**Authors:** Edenise Maria Santos da Silva Batalha, Elisabete Maria das Neves Borges, Marta Maria Melleiro

**Affiliations:** 1 Universidade do Estado da Bahia, Departamento de Ciências da Vida, Salvador, BA, Brasil.; 2 Escola Superior de Enfermagem do Porto, Porto, Portugal.; 3 Universidade de São Paulo, Escola de Enfermagem, São Paulo, SP, Brasil.

**Keywords:** Segurança do paciente, Satisfação pessoal, Esgotamento psicológico, Transtornos de estresse traumático, Enfermagem

## Abstract

**Objetivo:**

Analisar a associação entre cultura de segurança do paciente e qualidade de vida profissional de trabalhadores de enfermagem.

**Método:**

Estudo correlacional, realizado em um hospital de Salvador-BA, com 180 participantes. Os dados foram coletados por meio do *Hospital Survey on Patient Safety Culture e da Professional Quality of Life Scale* e analisados por testes de correlação.

**Resultados:**

O emprego do modelo Qualidade de Vida Profissional, que engloba a Satisfação por compaixão, o *Burnout* e o Estresse Traumático, permitiu verificar que melhor avaliação da cultura de segurança esteve associada negativamente ao *Burnout*. Referente às dimensões da cultura, melhores avaliações na percepção geral da segurança, trabalho em equipe e dimensionamento de pessoal associaram-se negativamente ao *Burnout* e ao Estresse Traumático. Maior *Burnout* associou-se negativamente à melhor passagem de plantão e maior Estresse Traumático positivamente à comunicação sobre erro.

**Conclusão:**

Maiores níveis de *Burnout* estiveram associados à pior percepção da cultura de segurança e piores avaliações acerca do trabalho em equipe; dimensionamento e percepção geral da segurança se associaram ao maior nível de *Burnout* e de Estresse Traumático, destacando a importância de investimentos nessas áreas.

## INTRODUÇÃO

O fortalecimento de uma cultura de segurança do paciente positiva, pautada na visão sistêmica do serviço de saúde, que prioriza a prevenção de incidentes e o aprendizado, é essencial para a garantia da qualidade na assistência. Essa cultura é reconhecida como uma das principais recomendações para a mitigação dos eventos adversos em saúde. Diante disso, a sua avaliação tem sido amplamente empregada nas instituições de saúde, propiciando identificar as dimensões da cultura vulneráveis e um diagnóstico acerca das necessidades de intervenção, bem como o direcionamento das ações de melhorias^(1,2)^.

O investimento na cultura de segurança do paciente, por meio da sua avaliação e de propostas de intervenção, requer o entendimento de que as atividades em saúde são desempenhadas essencialmente por pessoas e que a saúde física e psicológica dessas pessoas é fator primordial para o desenvolvimento e para o êxito das políticas de segurança. Ainda, é essencial compreender que o ambiente de trabalho e o seu grau de segurança podem influenciar na saúde e bem-estar dos trabalhadores. Assim, fortalecer a cultura de segurança do paciente requer a transversalização dessa área com outros aspectos organizacionais, especialmente com a saúde dos trabalhadores. Desse modo, a análise dessa relação pode evidenciar como outras áreas podem ser potencializadas, para que em conjunto entreguem resultados positivos para os pacientes e profissionais.

Nesse contexto, faz-se necessário refletir acerca do trabalho desenvolvido pelos profissionais da área da enfermagem, em virtude de serem a maior força de trabalho em saúde e prestarem cuidados diretos e contínuos aos pacientes, articulando o trabalho dos demais profissionais e assegurando a assistência^(3)^. Entretanto, pelas características inerentes do seu fazer laboral e diante das condições de trabalho aos quais estão submetidos, esses trabalhadores estão expostos a cargas físicas e psíquicas que podem interferir na sua saúde e bem-estar, o que pode influenciar na assistência^(4)^.

No cotidiano do seu trabalho, esses trabalhadores são expostos a fontes de sofrimento, seja pelo próprio processo de lidar com os paradoxos de vida e morte, saúde e doença ou pelas condições do ambiente de trabalho. Entre as fontes de sofrimento nesses trabalhadores, estão a falta de estrutura física e recursos materiais, associada à sobrecarga de trabalho e à falta de resolutividade desses problemas por parte dos gestores, além da morte de pacientes e da falta de comprometimento de alguns membros das equipes^(5)^. Ainda, muitas vezes, esses trabalhadores estão submetidos a um trabalho de alta intensidade, a condições de trabalho precárias e a falta de segurança nos processos e procedimentos na prática clínica^(3)^.

Todavia, apesar dos aspectos negativos citados, o trabalho proporciona vivências prazerosas, bem como a satisfação profissional e pessoal. Nesse sentido, os trabalhadores de enfermagem percebem como fontes de prazer no seu trabalho o reconhecimento por parte dos pacientes, a possibilidade de recuperação e melhora do quadro clínico e o bom relacionamento com a equipe^(5)^.

Fundamentada nessa dualidade de sentimentos positivos e negativos gerados e potencializados pelo trabalho de pessoas que se propõem a cuidar do outro como essência do seu fazer laboral, a psicóloga Beth Hudnall Stamm propôs o modelo que nomeou “Qualidade de Vida Profissional”, no qual estão incorporados esses dois aspectos, o positivo: Satisfação por Compaixão; e o negativo: Fadiga por Compaixão, que por sua vez se divide em *Burnout* e Estresse Traumático Secundário^(6)^.

A satisfação por compaixão refere-se ao prazer que o trabalhador sente ao realizar bem o seu trabalho, relacionado a poder ajudar os outros e sua capacidade de contribuir para o ambiente de trabalho. No que concerne a fadiga por compaixão, a primeira vertente diz respeito a sentimentos como exaustão e frustração, típicos do *Burnout*, associado a sentimentos de desesperança e dificuldades em lidar com o trabalho ou em fazer o trabalho de forma eficaz. A segunda vertente, o estresse traumático secundário, está relacionada à exposição a eventos estressantes extremos e traumáticos no trabalho, sendo caracterizada pelo medo e pelo trauma relacionado à assistência. Os efeitos negativos podem incluir dificuldades de sono pelo sentimento de medo, imagens intrusivas ou evitação de lembranças das experiências traumáticas do paciente^(6)^.

Diante das repercussões desses aspectos para os trabalhadores, organizações de saúde e pacientes, pesquisas vêm empregando esse modelo da qualidade de vida profissional^(7–9)^. Contudo, ainda são escassos os estudos que abordem a relação da qualidade de vida profissional com a cultura de segurança do paciente no contexto hospitalar brasileiro. A análise dessa relação é essencial para a compreensão da associação entre esses fatores e para o delineamento de estratégias combinadas provenientes das políticas de qualidade em saúde e segurança do paciente e de saúde do trabalhador.

Frente a essas considerações, esta investigação objetivou analisar a associação entre cultura de segurança do paciente e qualidade de vida profissional de trabalhadores de enfermagem. Dadas as características dos fenômenos estudados, testou ainda a hipótese de que melhores avaliações da cultura de segurança do paciente estariam associadas positivamente à satisfação por compaixão e negativamente ao *Burnout* e ao estresse traumático secundário.

## MÉTODO

### Desenho do Estudo

Trata-se de um estudo exploratório, transversal, correlacional e de abordagem quantitativa.

### Local

A pesquisa foi desenvolvida em um hospital público geral da região metropolitana de Salvador, no estado da Bahia, Brasil. A instituição, integrante da Rede Própria da Secretaria da Saúde do Estado, é composta por 640 leitos operacionais e configura-se como de grande porte, com perfil de alta complexidade, terciário e de caráter assistencial. Compõe a rede assistencial da Macrorregião Leste de Saúde do Estado da Bahia, prestando atendimento em regime ambulatorial referenciado e de internação, além da assistência hospitalar em caráter de urgência e emergência. O referido hospital é centro de referência em neurologia, além de ser referência para hemorragia digestiva, nefrologia, pediatria, clínica médica, cirurgia buco-maxilo, cirurgia geral, neurocirurgia, cirurgia pediátrica e neonatal, maternidade de alto risco, entre outras especialidades.

Diante da variedade de serviços ofertados pelo hospital, optamos por estruturar a coleta dos dados nas áreas de internação Médico-Cirúrgico e Materno-Infantil. Assim, as unidades nas quais ocorreu a coleta foram: Clínica Médica com 96 leitos; Clínica Cirúrgica com 107 leitos; Unidade de Terapia Intensiva Adulto com 20 leitos; Unidade de Terapia Intensiva Neonatal com 17 leitos e Obstetrícia com 60 leitos. Essas unidades somavam, portanto, no período da coleta, um total de 300 leitos operacionais ativos.

### População e Critérios de Seleção

A população do estudo foi composta pelos enfermeiros, técnicos e auxiliares de enfermagem que atuavam nas unidades supracitadas, nas quais ocorreu a coleta de dados, somando um total de 528 trabalhadores de enfermagem.

A amostragem foi por conveniência e os critérios de exclusão foram: trabalhadores com menos de 6 meses na instituição, que estivessem na ocasião da coleta de dados em período de férias, licenças e/ou afastamentos do serviço e que exercessem atividades exclusivamente administrativas. Após a aplicação dos critérios de exclusão, o número de trabalhadores elegíveis foi de 420.

### Definição da Amostra

Por se tratar de um estudo correlacional, a avaliação do tamanho da amostra considerou a correlação entre as variáveis em questão e adotou um coeficiente de correlação ≥ 0,25 como tamanho de efeito mínimo, um nível de signiﬁcância de 5% e um poder de teste de 90%^(10)^. Com base nesses parâmetros, a amostra coletada foi de 180 trabalhadores de enfermagem.

### Coleta de Dados

A coleta de dados ocorreu de janeiro a março de 2020 por meio de três questionários. O primeiro, denominado questionário socioeconômico e profissional, foi elaborado pelas autoras e objetivou a caracterização da amostra por meio de 12 questões, oito de múltipla escolha (p. ex.: sexo e categoria profissional) e quatro abertas para livre preenchimento (p. ex.: idade e tempo de experiência). O segundo foi o *Hospital Survey on Patient Safety Culture* (HSOPSC) da *Agency for Healthcare Research and Quality* (AHRQ)^(11)^, traduzido e validado para o Brasil^(12)^, e o terceiro foi o *Professional Quality of Life Scale 4 –* ProQol IV^(13)^, traduzido e validado para o Brasil^(14)^.

O HSOPSC apresenta 42 assertivas distribuídas em 12 dimensões, a saber: Dimensão (D)1: Trabalho em equipe dentro das unidades; D2: Percepção geral da segurança do paciente; D3: Respostas não punitivas aos erros; D4: Adequação de profissionais; D5: Aprendizado organizacional - melhoria contínua; D6: Expectativas e ações de promoção de segurança dos supervisores e gerentes; D7: Abertura da comunicação; D8: Retorno da informação e comunicação sobre erro; D9: Frequência de eventos relatados; D10: Passagem de plantão ou de turno/transferências; D11: Apoio da gestão para segurança do paciente; D12: Trabalho em equipe entre as unidades. Além do mais, avalia a nota de segurança do paciente e o número de eventos notificados pelos trabalhadores nos últimos 12 meses. O referido questionário tem incorporado em sua composição uma escala sociopsicológica com cinco níveis de medidas psicométricas (variando de Discordo Totalmente a Concordo Totalmente e de Nunca a Sempre), na qual, para fins de análise, a medida 1 é considerada pior avaliação e a 5, a melhor avaliação.

A ProQol IV, versão brasileira, é composta por 28 questões distribuídas pelos três componentes. Os itens da subescala de Satisfação por Compaixão abordam os benefícios do trabalho, sendo que suas questões tratam do orgulho, do sentimento de poder fazer a diferença na vida das pessoas, do entusiasmo e da satisfação por cuidar do outro. Enquanto a subescala de estresse traumático secundário se refere a fatores negativos da atividade profissional, abordando a tensão, o estresse e o trauma relacionados ao trabalho com pessoas em sofrimento. Portanto, engloba os efeitos danosos da exposição secundária a eventos estressantes. Por fim, a subescala de *Burnout* abarca aspectos ligados à exaustão emocional, ao sentimento de falta de energia e desânimo. A ProQol IV adota, também, uma medida escalar em cinco níveis de intensidade que varia de raramente a quase sempre com escores de 1 a 5.

Os trabalhadores foram convidados para a pesquisa em seu ambiente de trabalho, de forma presencial, sendo acordado o período para retorno do questionário preenchido. Esse período foi variável conforme a disponibilidade dos participantes. Aos trabalhadores, era dada a escolha de responder no mesmo momento em que eram convidados à pesquisa ou de levar o questionário consigo e devolver às pesquisadoras em outro momento. O tempo médio para responder aos três questionários foi de 20 a 25 minutos. A coleta foi realizada pela pesquisadora principal com o auxílio de três graduandas em enfermagem de uma universidade estadual da Bahia. Forneceram-se todos os esclarecimentos sobre a pesquisa e dúvidas foram sanadas pelas pesquisadoras tão logo apresentadas pelos participantes.

### Análise e Tratamento dos Dados

Os dados foram organizados e armazenados em planilhas do Microsoft Excel e a validação dos questionários para proceder às análises baseou-se em critérios estabelecidos pelas autoras dos instrumentos originais.

Os dados sociodemográficos e profissionais foram analisados por meio de estatística descritiva através da distribuição das frequências absoluta e relativa e as variáveis numéricas analisadas pelas frequências nos intervalos de classes e, também, pelo cálculo da média com seu respectivo desvio padrão.

Para alcançar o objetivo deste estudo e testar suas hipóteses, recorremos ao Teste de Correlação de *Spearman*. Dessa maneira, esse teste mensurou a associação de cada uma das dimensões da cultura de segurança do paciente, bem como da segurança do paciente total (calculada a partir da análise de todas as dimensões), da nota de segurança do paciente e do número de eventos notificados com a satisfação por compaixão, o estresse traumático secundário e o *Burnout*.

A normalidade residual foi avaliada por meio do Teste Shapiro-Wilk e a homoscedasticidade pelo Teste de Levene. O nível de significância adotado foi de 5% (grau de confiança de 95%) para todos os testes implementados. O programa estatístico R foi utilizado para a realização das análises.

### Aspectos Éticos

Esta pesquisa atendeu aos princípios éticos que envolvem as pesquisas com seres humanos determinados pela Resolução n°466/2012 do Conselho Nacional de Saúde. A pesquisa foi aprovada pelo Comitê de Ética em Pesquisa (CEP) da Escola de Enfermagem da Universidade de São Paulo (Parecer n°. 3.285.766, ano 2019) e pelo CEP do hospital – cenário do estudo (Parecer nº 3.731.330, ano 2019). Os participantes foram esclarecidos por meio do Termo de Consentimento Livre e Esclarecido, que foi entregue em duas vias idênticas assinadas pela pesquisadora responsável e pelo participante da pesquisa, ficando uma cópia com ambas as partes, garantindo a participação de forma autônoma, consciente, livre e esclarecida.

## RESULTADOS

### Caracterização dos Participantes da Pesquisa

A amostra foi composta por 180 trabalhadores de enfermagem, sendo 158 (87,8%) do sexo feminino com média de idade de 40 anos. O maior quantitativo foi de técnicos de enfermagem (n = 95; 52,8%) e o nível de escolaridade prevalente foi o ensino médio (n = 64; 35,8%). A maioria (n = 62; 34,4%) atuava nas Clínicas Cirúrgicas, possuía de 10 a 14 anos de experiência profissional (n = 47; 26,1%) e de 6 meses a 4 anos de experiência na instituição (n = 89; 50,0%).

A descrição completa dos dados sociodemográficos e profissionais encontra-se na Tabela 1.

**Tabela 1:** Caracterização dos participantes da pesquisa segundo as variáveis sociodemográficas e profissionais – Salvador, BA, Brasil, 2020.

Variáveis sociodemográficas e profissionais		N	%
**Sexo**		180	100,0
	Feminino	158	87,8
	Masculino	22	12,2
**Idade** (M = 40,0 DP = 8,4)		175	100,0
	19–28 anos	13	7,4
	29–38 anos	65	37,1
	39–48 anos	67	38,3
	Acima de 49	30	17,2
**Categoria Profissional**		179	100,0
	Auxiliar de Enfermagem	11	6,1
	Técnico de Enfermagem	95	52,8
	Enfermeiro	73	40,6
**Nível de Escolaridade**		179	100,0
	Médio Completo	64	35,8
	Graduação	49	27,4
	Especialização	47	26,3
	Mestrado	19	10,6
**Local de trabalho**		180	100,0
	Clínica Médica	37	20,6
	Clínica Cirúrgica	62	34,4
	UTIA	36	20,0
	UTIN	27	15,0
	Obstetrícia	18	10,0
**Experiência profissional** (M = 11,6 DP = 8,3)		180	100,0
	6 meses – 4 anos	41	22,8
	5–9 anos	40	22,2
	10–14 anos	47	26,1
	15–24 anos	34	18,8
	Acima de 25	18	10,0
**Tempo de experiência no serviço** **(**M = 7,0 anos DP = 6,6)		178	100,0
	6 meses – 4 anos	89	50,0
	5–9 anos	31	17,4
	10–14 anos	39	21,9
	Acima de 15 anos	19	10,7

Legenda: M: média; DP: desvio-padrão; UTIA: Unidade de Terapia Intensiva adulto; UTIN: Unidade de Terapia Intensiva Neonatal.

### Associação da Cultura de Segurança do Paciente e Qualidade de Vida Profissional

A satisfação por compaixão não se associou à cultura de segurança do paciente.

O *Burnout* apresentou correlação negativa significativa com a segurança do paciente total com um coeficiente de correlação (r) de –0,287 e valor de p de <0,001 e com a nota de segurança (r = –0,191; p = 0,01), demonstrando que, de formal geral, maiores índices de *Burnout* associaram-se negativamente à melhor avaliação da cultura de segurança do paciente. De maneira específica, maiores pontuações de Burnout estiveram associadas negativamente a cinco dimensões da cultura de segurança do paciente: D1: Trabalho em equipe dentro das unidades (r = –0,353; p < 0,001); D12: Trabalho em equipe entre as unidades (r = –0,322; p ≤ 0,001); D2: Percepção geral da segurança do paciente (r = –0,306; p ≤ 0,001); D4: Adequação de profissionais (r = –0,250; p ≤ 0,001); e D10: Passagem de plantão ou de turno/transferências (r = –0,148; p ≤ 0,05).

Quando analisada de forma geral, a cultura de segurança do paciente não esteve associada ao estresse traumático secundário. Porém, em se tratando das dimensões específicas, maiores índices de estresse traumático secundário também estiveram associados a piores avaliações das dimensões: D1: Trabalho em equipe dentro das unidades (r = –0,207; p ≤ 0,01); D2: Percepção geral da segurança do paciente (r = –0,188; p ≤ 0,05); D4: Adequação de profissionais (r = –0,193; p ≤ 0,01); e D12: Trabalho em equipe entre as unidades (r = –0,147; p ≤ 0,01). O estresse traumático secundário se associou positivamente a melhor avaliação na dimensão D8: Retorno da informação e comunicação sobre erro e o estresse traumático secundário (r = 0,16; p ≤ 0,05).

Os resultados completos estão dispostos na Figura 1. A referida figura revela, além dos valores do coeficiente de correlação, intervalo de confiança e significância, as cores referentes ao mapa de calor: correlações positivas são apresentadas em tons de vermelho e as negativas em tons de azul, sendo que, quanto mais escura for a cor, mais forte é a correlação entre as variáveis. Nota-se a predominância do tom de azul mais escuro na relação do *Burnout* com a cultura de segurança do paciente.

**Figura 1:** Análise das correlações entre as dimensões de Cultura de Segurança do Paciente, Nota de Segurança do Paciente, Número de Eventos Notificados e Segurança do paciente Total com a Satisfação por Compaixão, Estresse Traumático Secundário e Burnout – Salvador, BA, Brasil, 2020.

Legenda: Cor: correlação; 95% CI: Intervalo de confiança; *Valor-p <0,05; ** Valor-p <0,01; *** Valor-p <0,001. D1: Trabalho em equipe dentro das unidades; D2: Percepção geral da segurança do paciente; D3: Respostas não punitivas aos erros; D4: Adequação de profissionais; D5: Aprendizado organizacional – melhoria contínua; D6: Expectativas e ações de promoção de segurança dos supervisores e gerentes; D7: Abertura da comunicação; D8: Retorno da informação e comunicação sobre erro; D9: Frequência de eventos relatados; D10: Passagem de plantão ou de turno/transferências ; D11: Apoio da gestão para segurança do paciente; D12: Trabalho em equipe entre as unidades; Nota SP: Nota da segurança do paciente; EN: Número de eventos de segurança notificados nos últimos 12 meses; SPTotal: Segurança do Paciente Total; SC: Satisfação por Compaixão e ETS: Estresse Traumático Secundário.

## DISCUSSÃO

A cultura de segurança do paciente não esteve associada à satisfação por compaixão, resultado que divergiu do estudo prévio desenvolvido com enfermeiros em Portugal, no qual foi encontrado associação positiva significativa da satisfação por compaixão com quatro dimensões de cultura de segurança do paciente: Trabalho em equipe dentro das unidades; Percepção geral de segurança do paciente; Adequação de profissionais; e Aprendizado organizacional-melhoria contínua. Ainda, no estudo no contexto português, a nota de segurança do paciente esteve associada positivamente à satisfação por compaixão^(15)^. Alguns aspectos podem justificar esses achados, como as questões sociais e culturais, por serem países diferentes, com configurações distintas em relação ao sistema de saúde, à organização da área da enfermagem e ao próprio processo de trabalho, o que pode influenciar em como a satisfação é concebida e desenvolvida nos trabalhadores e em como relaciona-se com outros fatores organizacionais.

Apesar de nesta pesquisa a hipótese de que a satisfação por compaixão estaria positivamente associada à cultura de segurança do paciente não ter sido confirmada, faz-se necessário refletir acerca da sua importância. Um estudo com 10.305 enfermeiras coreanas identificou que a satisfação por compaixão tem um efeito mediador na relação entre estresse e *Burnout* e confirmou que, mesmo em uma situação estressante, um enfermeiro vivenciando a satisfação por compaixão pode contrabalançar a relação entre estresse e *Burnout*, resultando na redução do *Burnout* ^(16)^. Além disso, estudos identificaram que a satisfação por compaixão tem associação significativa negativa ao burnout e estresse traumático secundário^(7,8,14)^, demonstrando a magnitude do seu efeito positivo.

A associação negativa do *Burnout* com a cultura de segurança do paciente confirmou a hipótese deste estudo e é congruente com os resultados encontrados no estudo no contexto português^(15)^. Quando analisada de forma total, a cultura de segurança do paciente não apresentou associação com o estresse traumático secundário. Contudo, apresentou associação negativa com quatro dimensões da cultura de segurança e associou-se positivamente a uma delas, dados que confirmam parcialmente a nossa hipótese de pesquisa. Em Portugal, foi encontrada associação negativa do estresse traumático com três dimensões: Adequação de profissionais, Percepção geral da segurança do paciente e Resposta não Punitiva aos Erros^(15)^.

As associações observadas entre o *Burnout* e o estresse traumático secundário e a cultura de segurança do paciente podem ser avaliadas fazendo-se a alusão de que podemos estar diante de uma relação cíclica. Levando em consideração as características do *Burnout* e do estresse traumático secundário, os trabalhadores de enfermagem acometidos por essas alterações poderiam estar mais propensos ao menor cumprimento dos padrões de qualidade e, portanto, à entrega de um cuidado menos seguro. Além disso, poderiam apresentar mais dificuldades nas suas relações no trabalho, avaliando como pior a cultura de segurança do paciente. Secundariamente, por estarem em um ambiente de trabalho com baixa segurança para os pacientes, esses trabalhadores poderiam sofrer com os efeitos de uma cultura de segurança deficitária e apresentar níveis mais altos de *Burnout* e estresse traumático secundário^(15,17)^.

Especificamente em relação ao *Burnout* e à segurança do paciente, um estudo de revisão evidenciou que níveis mais altos de *Burnout* estiveram associados a níveis mais baixos de cultura de segurança do paciente, a menor frequência de notificações de incidentes de segurança, maiores índices de queda, de infecções relacionadas à assistência à saúde, de erros de medicação e de lapsos na adesão do controle de infecções. Níveis elevados de *Burnout* influenciavam na percepção da pressão no trabalho, o que esteve negativamente relacionado à segurança do paciente. Ademais, um melhor ambiente de trabalho foi diretamente associado a menores níveis de *Burnout*, o que posteriormente esteve relacionado a um maior nível de segurança do paciente e à mitigação de eventos adversos^(17)^.

Pesquisas continuam apontando para a relação negativa do *Burnout* com a cultura de segurança, demonstrando que trabalhadores em situações de grande demanda no trabalho e que vivenciam o *Burnout* apresentam maiores chances de avaliar negativamente a cultura de segurança do paciente^(18,19)^.

Voltando o olhar para as dimensões específicas que se associaram negativamente ao *Burnout* e ao estresse traumático secundário, percebe-se que esses aspectos exerceram maior influência na avaliação dos componentes da fadiga por compaixão. Além disso, os trabalhadores com pontuações mais altas e, possivelmente, mais alterações em seu estado de bem-estar pelo estresse traumático e/ou *Burnout* pontuaram mais negativamente nesses aspectos. Assim sendo, enfatizamos a necessidade de melhorias direcionadas a essas dimensões, conjugadas com a prevenção e mitigacão do *Burnout* e do estresse traumático secundário.

Quando se trata de melhorias na cultura de segurança do paciente de maneira geral, percebe-se um grande desafio no cenário brasileiro. Uma revisão de escopo analisou 36 pesquisas que empregaram HSOPSC em hospitais do Brasil e evidenciou que em 27 não houve dimensões fortalecidas nas instituições pesquisadas^(1)^. Esse dado demonstra o quanto ainda estamos distantes de vivenciar efetivamente uma cultura de segurança do paciente positiva nos hospitais e nos leva a refletir que essa cultura fragilizada pode ser fator desencadeador de mais sofrimento no processo de trabalho.

No contexto das estratégias que podem melhorar de forma geral a cultura de segurança do paciente, salienta-se a importância da educação permanente, por meio de programas de capacitação específicos, a fomentação de uma comunicação aberta no ambiente de trabalho, o incentivo à notificação de incidentes e a implementação de uma cultura não punitiva aos erros^(2)^.

A avaliação do trabalho em equipe na unidade e entre as unidades do hospital destacou-se na associação ao *Burnout* e ao estresse traumático secundário, demonstrando que, quanto mais o trabalhador percebia esse trabalho em equipe deficitário, mais pontuava em *Burnout* e estresse traumático secundário.

O trabalho em equipe possibilita a produção de melhores resultados na atenção à saúde e aumento da satisfação laboral, devendo ser incentivado como uma das estratégias para crescente complexidade das demandas dos pacientes e das organizações de saúde. Os atributos do trabalho em equipe são: comunicação, objetivos comuns, reconhecimento do trabalho de todos os membros da equipe, interdependência das ações, colaboração interprofissional e atenção centrada no paciente^(20)^. Diante dos achados, enfatiza-se a necessidade do investimento no trabalho em equipe, visando a potencialização de seus atributos no contexto de trabalho.

Melhores avaliações do dimensionamento de pessoal também foram associadas negativamente ao *Burnout* e ao estresse traumático. Esse resultado pode relacionar-se à associação conhecida entre um ambiente laboral com carga de trabalho excessiva e *Burnout* ^(6,21)^. O dimensionamento adequado propicia um ambiente de trabalho mais equitativo e proporciona mais oportunidades de a equipe desenvolver apoio mútuo, relações harmoniosas com respeito e suporte social entre os membros. Nessa reflexão, estudo apontou que o baixo suporte social pode predispor a maior risco de estresse traumático secundário^(22)^.

A partir da análise da força de correlação, o *Burnout* apresentou valores negativos de correlação mais fortes com a cultura de segurança do paciente, demonstrando que, ao aumentar o nível de *Burnout*, o trabalhador tendia a diminuir a sua avaliação positiva da cultura de segurança. Esses achados possivelmente dizem respeito às características do *Burnout*, uma vez que se apresenta de maneira mais progressiva e contínua, derivando de vivência de situações estressantes e conflituosas que podem levar à diminuição do comprometimento com o trabalho, redução da preocupação empática com os pacientes e sentimentos ruins em relação aos colegas, a si mesmo e à profissão escolhida^(6,21)^.

O *Burnout* está associado a frustrações e à fadiga no contexto em que o trabalho não corresponde mais às suas expectativas, gerando um sentimento de vazio, no qual ocorre uma ruptura significativa da identidade profissional do trabalhador, levando à diminuição do comprometimento e insatisfação, o que afeta o seu desempenho nas atividades laborais^(21)^. Em contraponto, o estresse traumático tem sua origem na traumatização do indivíduo pela possibilidade de sentir empatia pelo outro que sofre, sendo o processo empático um recurso responsável pelo envolvimento saudável e compassivo com as pessoas^(22)^. Para mais, o estresse traumático pode ser agudo e abrupto, ocorrendo, na maioria das vezes, uma recuperação mais rápida que o *Burnout* ^(6)^.

A dimensão sobre retorno da informação e comunicação sobre erro, que se refere ao fato de os trabalhadores serem informados sobre os erros que acontecem, receberem retorno sobre as mudanças implantadas e discutirem maneiras de prevenir erros, apresentou associação positiva significativa com o estresse traumático secundário. Estudo demonstrou que profissionais que vivenciam erros no seu processo de trabalho apresentam sintomas relacionados à traumatização^(23)^. É válido frisar que os profissionais reconhecem esses sintomas, mas, também, afirmam que a melhor forma de lidar com a ocorrência dos erros é por meio da comunicação e discussão (*debriefing*) com toda a equipe envolvida. Além disso, profissionais envolvidos em ocorrências de erros afirmam que conversar com os colegas sobre o erro e com os pacientes e familiares também é essencial^(23)^. Essa análise demonstra a importância da comunicação sobre erro, como princípio fundamental para o fortalecimento da cultura de segurança do paciente e recuperação do bem-estar dos profissionais.

Embora possuam características diferentes e tenham se relacionado de maneira distinta com a cultura de segurança do paciente, medidas que visem diminuir de forma combinada o *Burnout* e o estresse traumático secundário são essenciais para promoção da saúde do trabalhador e para a segurança do paciente. Essas intervenções são importantes, pois estudos demonstram a associação positiva significativa entre esses dois aspectos da fadiga por compaixão^(7,8,14)^. Destarte, algumas medidas podem ser implementadas, como a condução de intervenções baseadas em meditação, respiração e exercícios de autocompaixão durante os turnos de trabalho^(9)^, que se mostram eficazes para melhorar os índices de satisfação por compaixão e para reduzir a fadiga por compaixão.

Ademais, estratégias específicas para a prevenção e alívio do *Burnout* tornam-se essenciais e devem ser empregadas em nível individual, coletivo/grupal e organizacional. Do ponto de vista de estratégias individuais, os trabalhadores podem ser encorajados a desenvolverem a autoavaliação e autoconhecimento, além da adoção de hábitos mais saudáveis. Em nível coletivo/grupal devem ser promovidas estratégias que fomentem a interajuda, o compartilhamento dos sentimentos e percepções com a equipe, a construção de coletivos de trabalho fortes e com apoio mútuo. As intervenções em nível organizacional centram-se na oportunidade da participação ativa dos trabalhadores nas decisões laborais e na reestruturação das tarefas e das condições de trabalho, de modo a torná-las atrativas e gratificantes para os trabalhadores^(24)^.

Apesar de apresentar contribuições significativas à área da segurança do paciente e saúde do trabalhador, este estudo apresenta algumas limitações. A primeira se refere ao fato de ter sido desenvolvido em um cenário hospitalar específico, com uma amostra restrita de trabalhadores de enfermagem, pelo que a generalização dos resultados deve ser feita com cautela. A outra diz respeito ao fato de esta pesquisa ter sido desenvolvida apenas com a equipe de enfermagem, não apresentado resultados referentes a outras categorias profissionais.

A despeito dessas limitações, o estudo apresenta-se como inovador, contribuindo para melhorias na assistência e no gerenciamento dos serviços de enfermagem e de saúde, pois aponta para a necessidade de reestruturação das políticas organizacionais de segurança do paciente e saúde do trabalhador, com ênfase especialmente no trabalho em equipe e no dimensionamento adequado de pessoal, e na prevenção e alívio do *Burnout*.

## CONCLUSÃO

A partir da análise da associação da cultura de segurança do paciente com a qualidade de vida profissional, verificou-se que a satisfação por compaixão não se associou à cultura de segurança do paciente, mas, apesar disso, diante dos seus efeitos positivos, esta deve ser estimulada nos profissionais de saúde. Em se tratando da fadiga por compaixão, ratificou-se a relação negativa do *Burnout* com uma melhor avaliação da cultura de segurança do paciente, demonstrando que trabalhadores com maiores níveis de *Burnout* tendiam a avaliar como pior a segurança do paciente na unidade de trabalho e no hospital como um todo, o que evidencia a necessidade de intervenções que visem o fortalecimento de um cuidado mais seguro e a prevenção e alívio do *Burnout*, combinando estratégias em nível individual, coletivo e organizacional. Piores avaliações nas dimensões referentes ao trabalho em equipe, dimensionamento de pessoal e da percepção geral da segurança se associaram ao maior nível de *Burnout* e de estresse traumático, destacando a importância de maior investimento nessas áreas.

Diante dessas considerações, este estudo possibilitou verificar como esses aspectos relacionam-se e o quanto é factível a avaliação de forma conjugada para implementação de melhorias que beneficiem tanto os pacientes quanto os profissionais e, consequentemente, os serviços de saúde.

Por fim, acredita-se que esta investigação possa abrir caminhos para novas pesquisas, inclusive, com modelos de estudos longitudinais para acompanhamento e análise dos aspectos e sua associação ao longo do tempo, permitindo o aprofundamento na compreensão dessa relação e construção de novas propostas de intervenção.
